# Bilingual children weigh speaker’s referential cues and word-learning heuristics differently in different language contexts when interpreting a speaker’s intent

**DOI:** 10.3389/fpsyg.2015.00796

**Published:** 2015-06-10

**Authors:** Wan-Yu Hung, Ferninda Patrycia, W. Q. Yow

**Affiliations:** Humanities, Arts and Social Sciences, Singapore University of Technology and Design, SingaporeSingapore

**Keywords:** mutual exclusivity, bilingualism, code-switching, word-learning, communicative signals, pragmatic cues

## Abstract

Past research has investigated how children use different sources of information such as social cues and word-learning heuristics to infer referential intents. The present research explored how children weigh and use some of these cues to make referential inferences. Specifically, we examined how switching between languages known (familiar) or unknown (unfamiliar) to a child would influence his or her choice of cue to interpret a novel label in a challenging disambiguation task, where a pointing cue was pitted against the mutual exclusivity (ME) principle. Forty-eight 3-and 4-years-old English–Mandarin bilingual children listened to a story told either in English only (No-Switch), English and Mandarin (Familiar-Switch), English and Japanese (Unfamiliar-Switch), or English and English-sounding nonsense sentences (Nonsense-Switch). They were then asked to select an object (from a pair of familiar and novel objects) after hearing a novel label paired with the speaker’s point at the familiar object, e.g., “Can you give me the *blicket*?” Results showed that children in the Familiar-Switch condition were more willing to relax ME to follow the speaker’s point to pick the familiar object than those in the Unfamiliar-Switch condition, who were more likely to pick the novel object. No significant differences were found between the other conditions. Further analyses revealed that children in the Unfamiliar-Switch condition looked at the speaker longer than children in the other conditions when the switch happened. Our findings suggest that children weigh speakers’ referential cues and word-learning heuristics differently in different language contexts while taking into account their communicative history with the speaker. There are important implications for general education and other learning efforts, such as designing learning games so that the history of credibility with the user is maintained and how learning may be best scaffolded in a helpful and trusting environment.

## Introduction

Existing research in developmental psychology has shown that children use various strategies such as word-learning heuristics to help narrow down potential objects when trying to identify referents (Clark, 1988; Markman and Wachtel, 1988; Markman, 1994; Landau et al., 1998). An example of a word-learning heuristic is the *mutual exclusivity (ME) principle* that assumes a one-to-one correspondence between a label and an object, such that a novel label refers to a novel object rather than a familiar object (ME hereafter; Markman and Wachtel, 1988). Children by the age of two are also able to use a speaker’s cues, such as point and gaze, as clues to understanding the speaker’s referential intents (e.g., Lempers, 1979; Leung and Rheingold, 1981; Baron-Cohen, 1989; Franco and Butterworth, 1996; Povinelli et al., 1997). More recently, research has shown that a change in language contexts, such as code-switching (the alternate use of two languages in a single discourse), can heighten bilingual children’s use of a speaker’s point and gaze when determining a target referent object (Yow and Hung, 2013).

Research suggests that language environment such as growing up bilingually may affect how children weigh the importance of ME to understand referential intents, and that ME is typically more relaxed among bilingual children than monolingual children (Davidson et al., 1997; Byers-Heinlein and Werker, 2009; Houston-Price et al., 2010). By comparing infants from different language backgrounds, Byers-Heinlein and Werker (2009) found that infants from a monolingual background showed the strongest use of ME (e.g., looking significantly longer at an unknown object than at a familiar object when a novel label was used), followed by infants from a bilingual background and, finally, infants from a trilingual background, who showed the weakest use of ME (see Davidson et al., 1997, for a similar pattern in bilingual and monolingual preschool children). Other studies also found that when ME was pitted against other referential cues, such as pointing, bilingual children were more likely to violate ME in favor of pointing, as opposed to monolingual children who showed a more robust use of ME (Jaswal and Hansen, 2006; Yow and Markman, 2007; Healey and Skarabela, 2008; but cf. Grassmann and Tomasello, 2010).

There have been theoretical attempts to explain why bilingual children are more willing to suspend ME in the presence of other referential cues. One account suggests that living in a bilingual environment involves frequent encounter of situations where the same object can be named differently in different languages (e.g., *house* in English vs. *casa* in Spanish). It is believed that such experiences violate the assumption of the one-to-one word-referent mapping in ME and hence bilingual children are likely to perceive ME as a less helpful word-learning heuristic compared with referential cues such as pointing (Au and Glusman, 1990; Davidson et al., 1997; Healey and Skarabela, 2008). Another account suggests that bilingual children are flexible in perspective-taking and are adept at taking another person’s referential cues to learn about a novel situation or determine a target referent when there is a conflict with their own assumptions such as ME (Genesee et al., 1975; Rosenblum and Pinker, 1983; Healey and Skarabela, 2008). Thus, this bilingual advantage of perspective-taking is largely due to the demands of living in a bilingual environment, which requires assimilation and accommodation of different linguistic perspectives that are unique to individual languages (e.g., every French noun has a grammatical gender but English nouns have no grammatical gender associated with them). For instance, Bassetti (2007) found that while Italian monolingual children tended to attribute female voices for objects that are feminine in Italian, Italian–German bilingual children tended to hold more balanced views toward object gender, especially for objects with conflicting grammatical gender in different languages (e.g., *clock* is masculine in Italian but feminine in German).

We propose that bilingual children’s inclination to suspend ME could also be related to their frequent encounters with complex conversations in either language and code-switching (the alternate use of two or more languages in the context of a single conversation). Bilingual children have to often figure out what language a speaker is using and how to interact appropriately to avoid a potential communication breakdown. They may pay greater attention to a speaker’s referential cues (e.g., point and gaze) to determine the speaker’s communicative intent (Yow and Markman, 2011; Brojde et al., 2012). Bilingual children may thus rely more on a speaker’s referential cues than general word-learning heuristics (e.g., ME) to determine the speaker’s referential intent in a challenging communicative context. Yow and Hung (2013) found that bilingual preschoolers who heard a speaker code-switched in a mixture of known and unknown languages were better able to utilize the speaker’s point and gaze than those who did not, possibly to accommodate the extra communicative demands. Our study seeks to examine how an exposure to a code-switching scenario would influence bilingual children’s use of referential cues and ME in a challenging context where these two cues are pitted against each other.

Research has shown that exposure to code-switching may influence word learning and language processing in a number of aspects, such as receptive vocabulary (e.g., Byers-Heinlein, 2013), speed of lexical access (e.g., Macnamara, 1967; Grosjean, 1988), reading comprehension (e.g., Beauvillain and Grainger, 1987; Thomas and Allport, 2000), and naming and reading aloud (e.g., Kolers, 1966; Meuller and Allport, 1999). Nonetheless, the existing studies on this topic are predominantly about code-switching in languages that are spoken in one’s family (i.e., *familiar* code-switching). Little is known about how such language processes may be influenced by exposure to languages that are unknown to the listener (i.e., *unfamiliar* code-switching). This distinction between *familiar* and *unfamiliar* code-switching from a listener’s perspective could be important because there may be significant differences in the efforts required for comprehending these two types of code-switching. In the present study, we distinguished code-switching as either familiar or unfamiliar from the listener’s point of view (i.e., switch between languages known to the listener, or switch between a language known to the listener and another language unknown to the listener, respectively). As unfamiliar code-switching involves a language unknown to the listener, it is likely to incur some form of communication breakdown. The extent of efforts required for comprehending unfamiliar code-switching may be much greater than comprehending familiar code-switching. Therefore, unfamiliar code-switching may trigger children to pay attention to other referential cues (e.g., point) than solely depend on language-related heuristic, such as ME, in a conflicting situation.

To date, it remains relatively unknown whether these two types of code-switching would influence children’s word-learning in a context where both referential cues and ME are available. This study attempts to address this question by using a word disambiguation task to examine bilingual children’s choice of cue (ME or point) under different code-switching conditions. English–Mandarin bilingual children first heard a storytelling episode either in English only (No-Switch), English and Mandarin (Familiar-Switch), or English and Japanese (Unfamiliar-Switch), followed by a disambiguation task, where pairs of familiar and novel objects were presented to them and an experimenter requested for an object using a novel label while pointing to the familiar object. Since communication breakdown may be incurred in the unfamiliar code-switching condition but not the other two conditions, children may weigh and use the ME and point differently when trying to figure out the target referent. We predicted that children who heard unfamiliar code-switching would make use of the speaker’s point more than ME when interpreting the novel label compared to children who heard familiar or no code-switching.

## Study 1

### Participants

Thirty-six 3- and 4-years-old English–Mandarin bilingual children from three different childcare centers in Singapore participated in this study (17 females, 19 males; *M_age_* = 3;11, range 3;0–4;10). Prior to the experiment, parents filled a language background questionnaire that asked about their general demographic information and their children’s language use at home (see **Table 1**). Children were randomly assigned to one of three code-switching groups: No-Switch, Familiar-Switch, or Unfamiliar-Switch (see section on Storytelling), with the constraint that in the Unfamiliar-Switch group, we only included children who did not have any exposure to Japanese language to ensure that the children were indeed unfamiliar with the language.

**Table 1:** Demographic information and language use: means (SD).

	No-Switch	Familiar-Switch	Unfamiliar-Switch
Age	3;11 (0;7)	4;1 (0;8)	3;10 (0;8)
SES (Father)^a^	3.67 (0.99)	3.50 (0.67)	3.42 (0.67)
SES (Mother)^a^	3.50 (1.17)	3.50 (0.67)	3.42 (0.67)
Exposure to English (%)^b^	58.67 (20.64)	65.00 (12.25)	62.92 (16.58)
Exposure to Mandarin (%)^b^	34.17 (20.76)	32.08 (11.57)	29.42 (11.62)
Parental code-switching^c^	2.71 (0.40)	2.34 (0.54)	2.43 (0.38)
Working memory^d^	4.75 (1.36)	5.83 (1.34)	5.08 (1.44)
Inhibitory control^e^	13.08 (3.73)	13.58 (2.84)	12.75 (2.53)

*Total N = 36. Seven children had exposure to a third language for at least 10% of their time, which includes Tamil (n = 1), Hindi (n = 1), Malay (n = 1), Thai (n = 1), Japanese (n = 1), Cantonese (n = 1), and Hokkien (n = 1). These children were distributed across the three conditions*.

*^a^SES = Socioeconomic status measured by education level, in a range from 0 (none or no formal education) to 5 (postgraduate degree)*.

*^b^Average amount of exposure in a typical week in percentage*.

*^c^Two parents did not complete this questionnaire (one from the No-Switch condition and one from the Unfamiliar-Switch condition)*.

*^d^Working memory was measured by the forward digit-span test*.

*^e^Inhibitory control was measured by the day–night Stroop task*.

### Materials

#### Parents’ Code-Switching Questionnaire

This questionnaire was used to obtain information about parents’ code-switching behavior during their daily communication with the child. It contained eight items and was constructed based on the *Bilingual Switching Questionnaire* (Rodriguez-Fornells et al., 2012). The items asked parents how frequently they code-switch both in general and within a sentence, how frequently they code-switch for certain topics or issues, and how frequently they think they unintentionally code-switch during their conversation with their child. For each item, parents were asked to rate on a 5-point frequency scale (1 = *never* to 5 = *always*). A mean score of these items was calculated for each child.

#### Picture Books

We created two A5 size wordless color picture books (pictures were modified from de Bezenac, 2010a,b; http://www.freekidsbooks.org). Each picture book consisted of five pictures printed on five separate pages. The two picture books were matched on contents and the number of characters involved.

#### Objects and Labels

Six pairs of objects and six novel labels were used in the disambiguation task (see Appendix A). Each pair consisted of a familiar object and a novel object of similar size and of comparable visual attractiveness.

#### Forward Digit Span Task

This task was adapted from the Wechsler Intelligence Scale for Children-Revised (Wechsler, 1974) and was used to ensure that children in the three conditions were comparable in their working memory capacity. In this task, an experimenter read out a string of digits one at a time, and the child was asked to repeat them in the same order as the experimenter had recited them. The length of the digit strings started from two and increased by one digit after every two trials. The trials continued until two consecutive errors were made in trials of the same digit length. A list of 16 strings of digits was used and the longest string consists of eight digits. The total score reflected the number of strings the child repeated correctly, and ranged from 0 to 16.

#### Day–Night Stroop Task

We used the day–night Stroop task (adapted from Gerstadt et al., 1994) to ensure that children from the three conditions did not differ in inhibitory control capacity. We presented each child with a series of cards in a pre-determined random order (Siegal et al., 2009), each with either a picture of a moon or a sun on it. The child was instructed to say “day” on seeing a moon card and “night” for a sun card. There were two practice trials, followed by 14 test trials. The experimenter explained the rule again and restarted with the first two trials if the child failed either of the first two trials. Once the child successfully answered both practice trials, the experimenter continued to administer the remaining 14 trials. The total score ranged from 2 to 16.

### Procedure

This study was approved by Institutional Review Board (IRB) of the Singapore University of Technology and Design (SUTD). Children whose parents had given informed consent for their participation were tested individually in a quiet room at their childcare center. Each of them received a session of storytelling, a disambiguation task, a forward digit span task, and a day–night Stroop task, in this order.

#### Storytelling

An experimenter first introduced the child to one of the two wordless picture books by saying, “Look at this picture book! I am going to tell you a story.” She then proceeded with a story that consisted of five sentences, which corresponded to each of the five pictures in the picture book. For the No-Switch group, the experimenter told the story completely in English. For the Familiar-Switch group, the experimenter alternated the descriptions of the pictures in English and Mandarin (i.e., in this sequence: English–Mandarin–English–Mandarin–English). For the Unfamiliar-Switch group, the experimenter alternated the descriptions in English and Japanese in the same sequence as in the Familiar-Switch group. All the sentences were of comparable length (see Appendix B). The experimenter presented the picture book in front of the child on the table they shared, and helped turn the pages without pointing to any part of the pictures so as not to prime the child to attend to the experimenter’s point in the subsequent disambiguation task. The experimenter did not provide any feedback to the child throughout the storytelling episode. The child was given sufficient time to glance through the picture on each page before the experimenter continued to the next page.

#### Disambiguation Task

**FIGURE 1:**
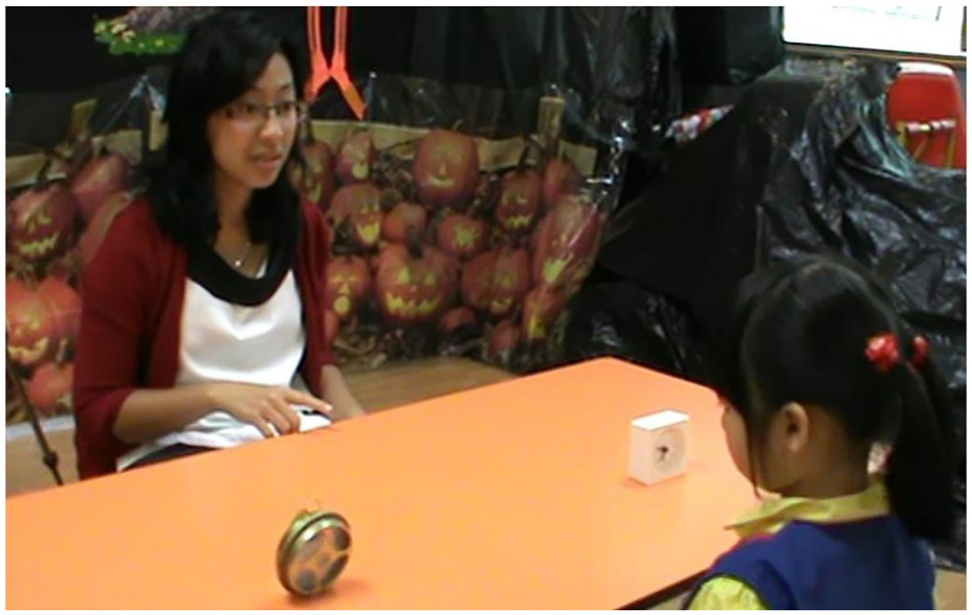
**An experimenter pointing to the familiar object of a pair of familiar and novel objects (familiar object: clock; novel object: mosquito coil)**.

After the storytelling session, the same experimenter conducted six trials of the disambiguation task adapted from Jaswal and Hansen’s (2006) procedures. For each trial, the experimenter first presented the child with a pair of one familiar object and one novel object, and directed the child’s attention to both objects equally without labeling them (e.g., “Look at these!”). The experimenter then placed the two objects on the table half way between herself and the child, slightly more than shoulder length apart, and asked the child to give her one of the objects by using a novel label, “Can you give me the *blicket*?” The task was made challenging by the experimenter pointing subtly but unambiguously to the familiar object while providing the novel label (see **Figure 1**). To draw the child’s attention, the experimenter made a gentle tap on the table twice every time before making the request. The experimenter kept her gaze direction neutral by looking straight at the child until a response was made. We counter-balanced the pairings of novel labels and object pairs, and the presentation order of the novel labels. For half of the children, the task started with the familiar object on the left. For each child, the familiar objects appeared on the child’s left side half of the times.

### Results

One-way between-subjects Kruskal–Wallis tests confirmed that children of the three code-switching groups were matched on age, amount of exposure to English and Mandarin, parental education level, reported amount of parental code-switching with child, working memory, and inhibitory control, all *p*s > 0.10. Non-parametric tests were used because the scores of the control variables were not normally distributed.

We hypothesized those bilingual children who heard unfamiliar code-switching would likely use the speaker’s point more often than children who heard familiar or no code-switching to interpret the novel label. Thus, responses of the disambiguation task were coded as “1” if the child chose the familiar object according to the experimenter’s point, or “0” if the child used ME to choose the novel object instead. The total score across the six trials ranged from 0 to 6.

A one-way between-subjects ANOVA showed that the three groups of children performed differently in the disambiguation task [*F*(2,33) = 4.33, *p* = 0.021, Cohen’s *d* = 1.73]. Bonferroni *post hoc* comparisons revealed that there was a significant difference between the Familiar-Switch and Unfamiliar-Switch groups, but not between the No-Switch and Familiar-Switch groups, or between the No-Switch and Unfamiliar-Switch groups (see **Table 2**). This suggests that bilingual children’s choice of cue (ME or point) was not influenced by the presence of code-switching *per se*, but rather by the *type* of code-switching used to communicate with them. Contrary to our hypothesis, the Unfamiliar-Switch group was less likely than the Familiar-Switch group to use the experimenter’s point to interpret the novel label. Two-tailed one-sample *t*-tests showed that while the No-Switch group performed at chance level [*t*(11) = -0.17, *p* = 0.87, Cohen’s *d* = -0.048], the Familiar-Switch group tended to use the experimenter’s point over ME to disambiguate the novel label, [*t*(11) = 1.82, *p* = 0.097, Cohen’s *d* = 0.53], and the Unfamiliar-Switch group significantly chose ME rather than the experimenter’s point above chance when disambiguating the novel label, [*t*(11) = -2.57, *p* = 0.026, Cohen’s *d* = -0.74]. A closer look at the distribution of children’s responses revealed that across the six trials, 33.3% of the No-Switch group used ME and point equally (three trials each), 41.7% used mostly ME (in four or more trials), and 25% used mostly point (in four or more trials). On the other hand, 8.3% of the Familiar-Switch group used ME and point equally, 25% used mostly ME and 66.7% used mostly point. In contrast, 41.7% of the Unfamiliar-Switch group used ME and point equally, 50% used mostly ME, and only 8.3% used mostly point.

**Table 2:** Average frequency of accepting the pointing cues to pick familiar objects (out of six trials).

Condition	Mean	SD
No-Switch	2.92	1.73
Familiar-Switch	4.08	2.07
Unfamiliar-Switch	2.00	1.35

*Total N = 36*.

An additional analysis was conducted on the children’s looking time toward the experimenter during the storytelling session. If extra efforts were required to comprehend the foreign sentences, we would expect the Unfamiliar-Switch group to look at the experimenter for interpretation more often than the other groups when the code-switched sentences were uttered. We calculated how long a child spent on looking at the experimenter when she code-switched. Two independent coders coded oﬄine the proportion of time a child looked at the experimenter when the second and fourth sentences were uttered, i.e., where instances of code-switching took place for the Familiar-Switch and Unfamiliar-Switch groups (inter-rater reliability *r* = 0.99, *p* < 0.001). Looking time of one participant from the Unfamiliar-Switch group and one from the No-Switch group could not be coded due to technical problems during recording. A one-way between-subjects ANOVA showed that the three groups were significantly different in their looking time [*F*(2,31) = 7.59, *p* = 0.002, Cohen’s *d* = 1.65], with the Unfamiliar-Switch group showing the longest look (*M* = 43.12%, SD = 25.95%), followed by the Familiar-Switch group (*M* = 19.14%, SD = 16.00%), and the No-Switch group (*M* = 12.01%, SD = 15.64%). Bonferroni *post hoc* comparisons revealed that the looking time difference was significant between the Unfamiliar-Switch and Familiar-Switch groups and between the Unfamiliar-Switch and No-Switch groups, but not between the Familiar-Switch and No-Switch groups. This finding reveals that the Unfamiliar-Switch group indeed paid more attention to (i.e., looked longer at) the experimenter when they heard unfamiliar code-switching compared to the Familiar-Switch and No-Switch groups. This supports our speculation that children in the Unfamiliar-Switch group are looking for some clarification or assistance from the experimenter when they do not understand the foreign utterances. It is to be noted that the experimenter in our study remained focused on telling the story based on her scripts and did not respond to the children at all. If children had expected the experimenter to clarify or provide clues to her foreign utterances but were “ignored” (i.e., experimenter did not respond), then it is possible that the Unfamiliar-Switch group subsequently chose to use ME to determine the referent in the disambiguation task as they believed that the experimenter’s point would not be helpful anyway.

In summary, this study showed that the type of code-switching differentially influenced children’s choice of cue (ME or point) in a disambiguation task. Unexpectedly, bilingual children in the Unfamiliar-Switch condition showed a significant tendency to use ME over the point compared to bilingual children in the Familiar-Switch condition. Children’s increased looking time to the experimenter during the unfamiliar code-switched sentences implied that they might have expected the experimenter to clarify her utterances when they did not understand her. Hence, when the experimenter failed to repair the breakdown in the communication during the storytelling session, children in the Unfamiliar-Switch condition might have subsequently chosen to rely on other strategies (i.e., ME) instead of her point to interpret the novel label in the disambiguation task. Nonetheless, it is also possible that this preference could be due to an abrupt phonological change involved in unfamiliar code-switching. The sudden change in phonological makeup of the utterances may have prompted children to default to word-learning heuristics to select a referent. To tease apart these two possibilities, Study 2 used a nonsense English storytelling condition to induce comparable semantic barriers as unfamiliar code-switching. If communication barriers dictated children’s performance, we predicted that children who heard nonsense English would similarly choose to rely on ME over the speaker’s point to interpret the novel label as those in the Unfamiliar-Switch group in Study 1. Alternatively, if the type of code-switching provides a unique communicative signal over and beyond semantic familiarity and comprehension, the two groups would differ in their choice of cues in the disambiguation task.

## Study 2

### Participants

Twelve other 3- and 4-years-old English–Mandarin bilingual children from the same childcare centers as Study 1 participated in this study (six females, six males; *M_age_* = 4;0, range = 3;6–4;10; see **Table 3**).

**Table 3:** Demographic information and language use: means (SD).

	Nonsense-Switch(Study 2)	Unfamiliar-Switch(Study 1)
Age	4;0 (0;6)	3;10 (0;8)
SES (Father)	4.00 (0.43)	3.42 (0.67)
SES (Mother)	3.92 (0.67)	3.42 (0.67)
Exposure to English (%)	65.00 (14.62)	62.92 (16.58)
Exposure to Mandarin (%)	32.67(13.93)	29.42 (11.62)
Parental code-switching	2.31 (0.56)	2.43 (0.38)
Working memory	5.92 (0.79)	5.08 (1.44)
Inhibitory control	11.00 (4.71)	12.75 (2.53)

*Total N = 24. Five children had exposure to a third language for at least 10% of their time, which includes Tamil (n = 1), Malay (n = 1), Teochew (n = 1), Hokkien (n = 1), and other Chinese dialect (n = 1). These children were distributed across the two conditions*.

### Materials

The children were presented with the same materials as in Study 1, except that a similar but different picture book was used (pictures were modified from de Bezenac, 2010c; http://www.freekidsbooks.org). This picture book and the picture books used in Study 1 were matched on contents and the number of characters involved.

### Procedure

This study was approved by IRB of the SUTD. Study 2 followed the same procedure as Study 1 except that the story was told in alternate English and English-sounding nonsense sentences (Nonsense-Switch). The sentences were comparable to those sentences used in Study 1 in length (see Appendix B). The nonsense words were chosen from two nonsense poems, Jabberwocky (Carroll, 1872), and The Faulty Bagnose (Lennon, 1965).

### Results

Mann–Whitney *U* tests using Bonferroni adjusted alpha levels of 0.01 per test (0.05/8) showed that children in the Nonsense-Switch group in Study 2 did not differ significantly from the Unfamiliar-Switch group in Study 1 on all the control variables. Non-parametric tests were used because the scores of the control variables were not normally distributed.

An independent-samples *t*-test between the Nonsense-Switch group in Study 2 and the Unfamiliar-Switch group in Study 1 confirmed that there was no significant difference in performance between the two groups [*t*(22) = -1.22, *p* = 0.24, Cohen’s *d* = -0.50; see **Table 4**]. Two-tailed one-sample *t*-tests also found that the Nonsense-Switch group performed at chance level [*t*(11) = -0.30, *p* = 0.77, Cohen’s *d* = -0.086]. Recall that in Study 1, the Unfamiliar-Switch group significantly used ME more than the experimenter’s point [*t*(11) = -2.57, *p* = 0.026, Cohen’s *d* = -0.74]. This suggests that although overall, the Nonsense-Switch group did not differ significantly from the Unfamiliar-Switch group, they, in fact, used ME and the experimenter’s point equally often to interpret the novel label, compared to the Unfamiliar-Switch group who used ME over the experimenter’s point. A more detailed examination of the children’s responses revealed that 41.7% of the Nonsense-Switch group used ME and point equally (three trials each), 33.3% used mostly ME (in four or more trials out of six), and 25% used mostly point (in four or more trials of six). While 41.7% of the Unfamiliar-Switch group in Study 1 also used ME and point equally, 50% of them used mostly ME, and only 8.3% used mostly point.

**Table 4:** Average frequency of accepting the pointing cues to pick familiar objects (out of six trials).

Condition	Mean	SD
Nonsense-Switch (Study 2)	2.83	1.95
Unfamiliar-Switch (Study 1)	2.00	1.35


We also coded the proportion of time each child looked at the experimenter at both instances of code-switching (during the second and fourth sentence of the story). An independent-samples *t*-test revealed that the difference between the two groups were marginally significant [*t*(21) = 1.75, *p* = 0.094, Cohen’s *d* = -0.73; *M*_*Unfamiliar-Switch*_ = 43.12%, *SD*_*Unfamiliar-Switch*_ = 25.95%, *M*_*Nonsense-Switch*_ = 24.81%, *SD*_*Nonsense-Switch*_ = 24.19%). The Nonsense-Switch group tended to look less at the experimenter when they heard the nonsense sentences compared to the Unfamiliar-Switch group.

In summary, while children in the Nonsense-Switch group seemed to perform similarly as those in the Unfamiliar-Switch group in their choice of cue in a disambiguation task, their behavior was less consistent than the Unfamiliar-Switch group in relying on ME over the experimenter’s point. They also looked less at the experimenter when hearing the nonsense sentences compared to the Unfamiliar-Switch group when hearing the unfamiliar sentences. This result suggests that the unfamiliar code-switching effect found in Study 1 cannot be attributed to semantic barriers *per se*, and there is something unique about the communicative intent of a speaker when switching between familiar and foreign utterances.

## General Discussion

This research sought to answer whether exposure to a language switch, in particular, the specific *types* of switch, would influence bilingual children’s choice of cue (ME or point) in understanding referential intents. Our study showed that, indeed, the *type* of code-switching influenced the children’s choice of cue. The No-Switch and Nonsense-Switch groups were equally likely to use the experimenter’s point and ME to interpret a novel label. This finding of the No-Switch group was consistent with Yow and Markman (2007) where they found a proportionate use of the speaker’s point and ME among bilingual children in an analogous disambiguation task without prior episodes of code-switching. While the Familiar-Switch group showed a tendency to use the speaker’s point instead of ME, the Unfamiliar-Switch group significantly used ME instead of the speaker’s point. Although this seems to contradict our prediction that children who heard unfamiliar code-switching would pay more attention to a speaker’s referential cues to overcome communicative challenges and thus rely on the speaker’s point over ME to interpret a novel label, our analysis of the children’s looking time revealed otherwise. The Unfamiliar-Switch group did look at the experimenter significantly longer when they heard the unfamiliar code-switched sentences than those who heard only English sentences, familiar English–Mandarin sentences, or English and nonsense English sentences. This suggests that unfamiliar code-switching provides a distinctive signal in the communication process, possibly above and beyond the semantic difficulties in comprehension experienced in other types of language use (such as nonsense English words).

We reasoned that the bilingual children looked at the experimenter longer when hearing the unfamiliar code-switching because they were expecting the experimenter to provide some clarification to help them understand her utterances, or at least some cues as to what these unfamiliar utterances were about. This is because code-switching usually serves as a way to contextualize daily conversation, for example, in quoting someone (Gardner-Chloros et al., 2000), to acquire the conversational turn in overlap multiparty play episodes (Cromdal, 2001), or to mark topic changes and text-to-text connection during book-reading activities (Kabuto, 2010). It is likely that children in the Unfamiliar-Switch group were expecting the experimenter to contextualize the unfamiliar language switch during the storytelling episode.

Yet, the experimenter gave no feedback to the child and provided no explanation to the code-switched sentences throughout the storytelling episode. The Unfamiliar-Switch group might have perceived that the experimenter was unhelpful and unreliable because the communication breakdown was left unresolved. The Unfamiliar-Switch group might then assume that the experimenter’s point during the disambiguation task would not be helpful or reliable in interpreting the novel label after all. Studies have shown that children tended to judge a person as unhelpful or tended to avoid choosing the person as a source of help if the person had previously provided insufficient or incomplete information to them (Gweon et al., 2011; Gillis and Nilsen, 2013). Consistent with our results, Krogh-Jesperson and Echols (2012) also found that children’s willingness to accept second labels depended on the perceived credibility of the speakers. This could explain why the Unfamiliar-Switch group chose to use their own ME assumptions over the experimenter’s point to interpret the novel label instead, even though they have paid more attention to her earlier.

One possible interpretation of our results is that because the Unfamiliar-Switch group assumed the experimenter’s point would not be helpful, they chose to *avoid* following the referential cue rather than chose to use the ME principle. We argue that the Unfamiliar-Switch group was more likely to use the ME principle rather than choose to avoid using the cue because word-learning heuristics are robust assumptions that children use to help narrow down potential objects (e.g., Jaswal and Hansen, 2006). That said, further studies could tease these two possible interpretations apart. For example, a three-object-choice paradigm could be used with the disambiguation task instead of a two-object-choice, that is, children are asked to choose between two familiar and one novel objects. If children were using ME rather than avoiding the experimenter’s cue, then they would choose the novel object significantly more often than the other familiar object not pointed at. If children were avoiding following the experimenter’s cue rather than using ME, then they would be equally likely to choose the novel object or the other familiar object not pointed at.

Nevertheless, our studies showed that there are nuances in the use of different cues when trying to understand a speaker’s referential intent. Bilingual children are generally willing to relax ME and use the speaker’s point to label a familiar object with a novel name. But this strategy may change, depending on the social communication process bounded by the context of a language switch. Bilingual children may perceive the social cues of the speaker as unhelpful or unreliable if the speaker did not behave according to the social rules surrounding language use. In this case, children may default to using word-learning heuristics to select a referent instead. Earlier unresolved social communication challenges may impact on how the social cues given by the same person will be interpreted and used later.

Our study demonstrated how the same information (e.g., gesture) might be utilized differently based on the experiences people previously had (e.g., violation of social expectation). Children tend to return to their default learning strategy as compared to possibly more effective methods provided by the speaker if they perceive the speaker as not helpful. This provides important implications for other domains that involve interactions between people and even those that involve learning applications. For example, the initial trust between learners and learning software may be undermined with a few instances of violation of expectation. The entire learning process may then lose its projected effectiveness as the learner starts to perceive the software as not helpful or unreliable. Thus, learning software and learning games may have to be designed in such a way that their credibility with the user is not lost as learning strategy changes.

In summary, we found that bilingual children were selective in their choice of cue to interpret a novel label depending on the surrounding language context (e.g., familiar or unfamiliar code-switching). We argued that bilingual children pay increased attention to the speaker when hearing unfamiliar code-switching partly for the purpose of overcoming communication challenges. Despite this, we found that bilingual children did not necessarily use the speaker’s point to interpret a novel label. They would weigh the various sources of information available to them and rely more on their own ME assumptions if they regarded the speaker as unhelpful according to their past interaction with the speaker. Future studies could examine whether bilingual children would regard a speaker who code-switches in an unfamiliar language as an unhelpful informant, and how this perception of unhelpfulness might influence their willingness to accept the speaker’s communicative cues to interpret a novel label. Further studies could also examine how children’s perceived helpfulness of the speaker would generalize to other learning contexts, such as from adults vs. from educational software, pointing cues vs. paralinguistic cues, etc. This may have important implications on general education and how learning can be best scaffolded in a helpful and trusting environment.

## Conflict of Interest Statement

The authors declare that the research was conducted in the absence of any commercial or financial relationships that could be construed as a potential conflict of interest.
