# Supplementary material for: Bilingual children weigh speaker’s referential cues and word-learning heuristics differently in different language contexts when interpreting a speaker’s intent
Source: Front Psychol. 2015 Jun 10;6:796. doi: 10.3389/fpsyg.2015.00796 (PMC4461834; doi:10.3389/fpsyg.2015.00796)
Supplement: Supplementary file 1 [file Appendix_A_and_B.docx]

**Supplementary Material**

**Use of mutual exclusivity and pragmatic cues in children’s understanding of referential intents**

Wan-Yu Hung^1^, Ferninda Patrycia^1^, W. Quin Yow^1*^

^1^Singapore University of Technology and Design, Humanities, Arts, & Social Sciences, Singapore

***Correspondence**: W. Quin Yow, Singapore University of Technology and Design, Humanities, Arts, & Social Sciences, 8 Somapah Road, Singapore, 487372. [quin@sutd.edu.sg](mailto:quin@sutd.edu.sg)

**Appendix A**

Familiar-and-novel object pairs and novel labels

| *Familiar and Novel Object Pairs* | |  |
| --- | --- | --- |
| Familiar objects | Novel objects | |
| Water bottle | Hand gripper | |
| Ball | Pedicure finger holder | |
| Toothbrush | Bike locking tool | |
| Toy bus | Whistle toy | |
| Clock | Mosquito coil | |
| Cup | Toilet roll holder | |
|  |  | |
|  | |  |
| *Novel Labels* |  | |
| *peri, dawnoo, blicket, toma, cardle, gazer* |  | |

**Appendix B**

Storytelling scripts and the number of syllables per sentence

No-Switch condition (version 1)

*Sentence 1*. This is Peter and this is his dog, Max; they are good friends. (n = 14)

*Sentence 2*. One day, Peter’s mother makes some cookies and the cookies taste so good. (n = 17)

*Sentence 3*. The cookies look very nice; Peter wants to eat the cookies. (n = 15)

*Sentence 4*. Max likes to eat cookies too, so Peter shares the cookies with Max. (n = 16)

*Sentence 5*. This makes them happy; this is the end of the story (n = 13)

No-Switch condition (version 2)

*Sentence 1.* This is Susan and this is Tom; Susan and Tom are good friends. (n = 15)

*Sentence 2.* They go to school together and they like to play together too. (n = 16)

*Sentence 3.* Susan wants to play with Tom’s toys, they look really fun to play with. (n = 16)

*Sentence 4.* Tom likes to play with Susan and he shares his toys with her. (n = 14)

*Sentence 5.* This makes them happy; this is the end of the story. (n = 13)

Familiar-Switch condition

*Sentence 1*.This is Peter and this is his dog, Max; they are good friends. (n = 14)

*Sentence 2. 有一天彼得的媽媽做了好香的餅乾.* (n = 15)

*Sentence 3.* The cookies look very nice. Peter wants to eat the cookies. (n = 15)

*Sentence 4. 彼得高興的拿了餅乾, 分了些給皮皮.* (n = 15)

*Sentence 5.* This makes them happy; this is the end of the story. (n = 13)

Unfamiliar-Switch condition

*Sentence 1.* This is Susan and this is Tom; Susan and Tom are good friends. (n = 15)

*Sentence 2.* *Karera wa issoni asobu no ga suki des.* (n = 15)

*Sentence 3.* Susan wants to play with Tom’s toys, they look really fun to play with. (n = 16)

*Sentence 4.* *Kare wa Susan to omocha o wake atemas.* (n = 15)

*Sentence 5.* This makes them happy; this is the end of the story. (n = 13)

Nonsense-Switch condition

Sentence 1. This is John and his turtle, Bob. John and Bob are good friends. (n = 14)

*Sentence 2.* *John gimble wabe toves brillig twas mimsy borogoves*. (n = 13)

*Sentence 3.* Mom helps John to take care of Bob because Mom likes Bob too. (n = 14)

*Sentence 4.* *Mom mungle pilgriffs thee waysock gurled bagnose somforbe.* (n = 13)

*Sentence 5.* This makes them happy; this is the end of the story. (n = 13)
